# Aloe-emodin exhibits growth-suppressive effects on androgen-independent human prostate cancer DU145 cells via inhibiting the Wnt/β-catenin signaling pathway: an *in vitro* and *in silico* study

**DOI:** 10.3389/fphar.2023.1325184

**Published:** 2024-01-29

**Authors:** Talib Hussain, Ahmed Alafnan, Ibrahim Abdullah Almazni, Nawal Helmi, Afrasim Moin, Hanadi M. Baeissa, Amir Mahgoub Awadelkareem, AbdElmoneim O. Elkhalifa, Tahani Bakhsh, Abdulrahman Alzahrani, Rashed Mohammed Alghamdi, Mohammad Khalid, Rohit Kumar Tiwari, Syed Mohd Danish Rizvi

**Affiliations:** ^1^ Department of Pharmacology and Toxicology, College of Pharmacy, University of Ha’il, Ha’il, Saudi Arabia; ^2^ Department of Clinical Laboratory Sciences, Faculty of Applied Medical Sciences, Najran University, Najran, Saudi Arabia; ^3^ Department of Biochemistry, College of Science, University of Jeddah, Jeddah, Saudi Arabia; ^4^ Department of Pharmaceutics, College of Pharmacy, University of Ha’il, Ha’il, Saudi Arabia; ^5^ Department of Biological Science, College of Science, University of Jeddah, Jeddah, Saudi Arabia; ^6^ Department of Clinical Nutrition, College of Applied Medical Sciences, University of Hail, Ha’il, Saudi Arabia; ^7^ Department of Applied Medical Sciences, Applied College, Al-Baha University, Al-Baha, Saudi Arabia; ^8^ Department of Laboratory Medicine, Faculty of Applied College, Al-Baha University, Al-Baha, Saudi Arabia; ^9^ Department of Pharmacognosy, College of Pharmacy, Prince Sattam Bin Abdulaziz University, Al-Kharj, Saudi Arabia; ^10^ Department of Clinical Research, Sharda School of Allied Health Sciences, Sharda University, Gautam Buddh Nagar, India

**Keywords:** aloe-emodin, ROS, DU145 cells, caspases, mitochondrial viability

## Abstract

At the molecular level, several developmental signaling pathways, such as Wnt/β-catenin, have been associated with the initiation and subsequent progression of prostate carcinomas. The present report elucidated the anti-cancerous attributes of an anthraquinone, aloe-emodin (AE), against androgen-independent human prostate cancer DU145 cells. The cytotoxicity profiling of AE showed that it exerted significant cytotoxic effects and increased lactose dehydrogenase levels in DU145 cells (*p* < 0.01 and *p* < 0.001). AE also induced considerable reactive oxygen species (ROS)-mediated oxidative stress, which escalated at higher AE concentrations of 20 and 25 μM. AE also efficiently instigated nuclear fragmentation and condensation concomitantly, followed by the activation of caspase-3 and -9 within DU145 cells. AE further reduced the viability of mitochondria with increased cytosolic cytochrome-c levels (*p* < 0.01 and *p* < 0.001) in DU145 cells. Importantly, AE exposure was also correlated with reduced Wnt2 and β-catenin mRNA levels along with their target genes, including cyclin D1 and c-myc. Furthermore, the molecular mechanism of AE was evaluated by performing molecular docking studies with Wnt2 and β-catenin. Evidently, AE exhibited good binding energy scores toward Wnt2 and β-catenin comparable with their respective standards, CCT036477 (Wnt2 inhibitor) and FH535 (β-catenin inhibitor). Thus, it may be considered that AE was competent in exerting anti-growth effects against DU145 androgen-independent prostate cancer cells plausibly by modulating the expression of Wnt/β-catenin signaling.

## 1 Introduction

According to the recent data from the Global Cancer Observatory in 2020, prostate cancer patients contributed 7.3% to the global burden of cancer-related malignancies. Furthermore, prostate cancer was also correlated with 3.8% of cancer-associated fatalities globally (Global Cancer Observatory Factsheet, 2020). Prostate cancer is more frequent in older men during the latter half of their life, thus increasing its mortality and morbidity. In spite of substantial breakthroughs previously reported in the research on prostate carcinomas, the information elucidating its exact cause still remains elusive (Rawla, 2019). Nevertheless, certain important risk factors have been identified that regulate the onset and progression of prostate malignancies. Of these risk factors, impaired metabolism of androgen, ethnicity, and diet and mutated oncogenes are the most prominent ones. Indeed, subtle genetic mutations, even at a single amino acid or functional group level, initiate prostate carcinomas (Shukla and Gupta, 2004). Moreover, reports have further substantiated that impaired crosstalk between various signaling pathways is critical in increasing the invasiveness and proliferation of prostate cancer cells (Ehsani et al., 2021). Previous reports have indeed outlined that crosstalk between androgen receptor (AR), NF-κB, and Wnt/β-catenin signaling is imperative in increasing the metastasis and progression of prostate cancers (Lee et al., 2013; Koushyar et al., 2022).

Downstream molecules of the β-catenin pathway have also been reported to act as co-activators of the androgen receptor which itself is an oncogene associated with prostate carcinoma. Peptidyl-prolyl isomerase, also referred to as Pin1, is reported to influence the proliferation of prostate cancer cells (Chen et al., 2006). Moreover, it has also been reported that in the absence of androgens, the Wnt/β-catenin pathway modulates the switching on of the androgen-mediated transcription pathway (Seo et al., 2017). Intriguingly, impaired androgen activity has also been reviewed to elevate Wnt/β-catenin signaling which culminates into the promotion of the androgen-independent proliferation of prostate cancer cells (Li et al., 2017; Wang et al., 2021). The crosstalk between β-catenin and AR often leads to a progression of prostate carcinomas, due to altered gene expression. Intriguingly, reports have shown that hyperactivated Wnt signaling results in escalated levels of β-catenin within the nucleus, leading to the expression of tumor-promoting genes (Zhan et al., 2017). A recent report correlated Wnt3 regulation with reduced β-catenin levels in the nucleus, leading to impaired cell cycle progression within prostate cancer cells (Dong et al., 2020a). Another report also showed that the modulation of Wnt/β-catenin by microRNA imparts therapeutic effects in prostate cancer (Cui et al., 2019). Indeed, these observations, along with others, make Wnt/β-catenin signaling a plausible target for the therapeutic intervention of prostate cancer (Wang et al., 2018; Cui et al., 2019; Liu et al., 2019).

Reports have substantiated that apoptotic cell death is indeed regulated by the crosstalk between Wnt/β-catenin and this has significant implications in several diseases (Ma et al., 2023). Apoptosis is undoubtedly a critical mechanism of immune responsiveness. Wnt/β-catenin signaling regulates several apoptosis-related receptors including Fas and TRAIL, involved in the activation of the extrinsic pathway, as well as Bax/Bcl-2 involved in modulating intrinsic apoptosis pathways and caspases (Verbrugge et al., 2010).

Aloe-emodin (AE) is an anthraquinone that has been found abundantly in *Aloe barbadensis miller*, *Cassia occidentalis*, and *Polygonum multiflorum*. It is a well-known member of the anthraquinone family (Dong et al., 2020a). In addition to the various pharmacological properties, AE has also been known for its effects on cell cycle progression, apoptosis, immune signaling, and metastasis (Sanders et al., 2017; Dong et al., 2020b). Moreover, AE is also reported to induce apoptosis via intrinsic and extrinsic apoptotic pathways (Sanders et al., 2018). Nevertheless, the effects of AE on DU145 cells remain unexplored till date. Therefore, the investigators tried to study the anti-cancer effects of AE on DU145 cells and further explored AE-mediated modulatory effects on Wnt/β-catenin signaling.

## 2 Materials and methods

### 2.1 Materials

Cell culture reagents, including Ham’s F-12K media, fetal bovine serum (FBS), antibiotic–antimycotic cocktail, MTT stain, Hoechst 33342, and DCFH-DA dye, were procured from Sigma (St. Louis, MO, United States). RNase-A and the HiPurA TM Total RNA Miniprep Purification Kit used were from HiMedia, Mumbai, India. The DyNAmo ColorFlash SYBR Green Quantitative Polymerase Chain Reaction (qPCR) Kit was from Thermo Fisher Scientific, Waltham, MA, United States. The primers in the study were procured from IDT, Coralville, IA, United States.

### 2.2 Methods

#### 2.2.1 *In vitro* studies

##### 2.2.1.1 Cell culture

Androgen-independent human-derived prostate cancer DU145 cells and J774A.1 murine alveolar macrophages were procured from the National Centre of Cell Sciences, Pune, India. The cells were allowed to proliferate in RPMI 1640 and DMEM high-glucose media supplemented with 10% FBS and 1% antibiotic–antimycotic solution, both v/v. The cells were continuously provided a humidified atmosphere with 5% CO_2_ at 37°C. All the imaging reported in the present study was carried out using bright light and various fluorescent channels of the FLoid Imaging Station (Thermo Fisher Scientific, Waltham, Massachusetts, United States).

##### 2.2.1.2 Cytotoxicity analysis

As described previously, AE-induced cytotoxic effects on DU145 cells were evaluated using MTT stain (Tiwari et al., 2021). AE was dissolved in 0.5% dimethyl sulfoxide (DMSO). Initially, 1 × 10^4^ DU145 cells were exposed to 5, 10, 15, 20, and 25 μM concentrations of AE for 24 h; after exposure, 5 mg/mL MTT stain (10 μL) was added in cells treated with various AE concentrations and incubated for another 4 h. Eventually, the cells were analyzed for the formation of formazan crystal by measuring their intensity at 570 nm. The cytotoxicity of AE against DU145 cells was expressed as cell viability percentage (%) using the following formula.

Cell viability (%) = (absorbance of treated DU145 cells)/(absorbance of positive control-treated DU145 cells) × 100.

Cells treated with 0.5% DMSO served as vehicle control and were subsequently used to compare the cytotoxicity of AE in DU145 cells. Furthermore, the effect of AE on J774A.1 cells was also evaluated by calculating the cell viability of J774A.1 cells using the same formula mentioned above.

##### 2.2.1.3 Lactose dehydrogenase assay

The ability of AE to induce cytotoxicity against DU145 cells was also reaffirmed by lactose dehydrogenase assay (LDH) assay, as described previously (Ahmad et al., 2021). Initially, 5 × 10^3^ DU145 cells were exposed to 5–25 μM concentrations of AE for 24 h. The levels of LDH in the supernatant of cells treated with varying AE concentration was determined colorimetrically using ELISA. 100 μL of the reaction mixture was supplemented to the supernatant collected from respective AE-treated DU145 cells. The mixture was briefly incubated in the dark for an additional 30 min. The conversion of NAD^+^ to NADH, characterized by the forming of formazan was quantified by recording the absorbance of the supernatant at 490 nm with the help of a spectrophotometer (Bio-Rad, Hercules, CA, United States). The formazan levels formed during the reaction were directly proportional to the amount of LDH released. Cytotoxicity was expressed in percentage (%) using the following formula.

Cytotoxicity (%) = ((compound-treated LDH activity)-(spontaneous LDH activity))/(maximum LDH activity spontaneous LDH activity) × 100.

##### 2.2.1.4 AE-induced effects on nuclear morphology

Hoechst 33342 stain was used for qualitatively assessing the fragmentation of AE-treated DU145 cells as stated previously (Ahmad et al., 2021). For analysis, 5 × 10^3^ DU145 cells were treated with varying stated AE concentrations for 24 h. After that, the media of each well were replaced with 2 μg/mL of Hoechst 33342 stain, and the plate was briefly incubated for 10 min in a humidified condition having 5% CO_2_. The characteristic blue fluorescence of the Hoechst 33342 stain in AE-treated DU145 cells was visualized, recorded, and compared with the control cells to assess changes in nuclear morphology.

##### 2.2.1.5 AE-mediated effects on oxidative stress

Reactive oxygen species-mediated oxidative stress in AE-exposed DU145 cells was assessed through dichlorodihydrofluorescein diacetate (DCFH-DA) stain as per the previously described protocol (Alshehri et al., 2022). For this assay, approximately 1 × 10^3^ DU145 cells were exposed to 5–25 μM concentrations of AE for 6 h. Subsequently, 10 μM DCFH-DA was supplemented to each for an additional 30 min, and the plate was left undisturbed in the dark. After incubation, the cells for each group were carefully washed using 1X phosphate buffer saline (PBS). The cells were then visualized and recorded for DCF-DA-mediated green fluorescence. The intensity of the DCFH-DA fluorescence in AE-treated DU145 cells (at the stated concentration) was compared with that of the untreated control DU145 cells.

For quantitative assessment, the same protocol was followed, except that approximately 1 × 10^5^ DU145 cells were exposed to each stated concentration of AE for 6 h. Subsequently, after following nearly the same protocol, the cells were quantified for their DCFH-DA-related median fluorescence intensity (MFI). The DCFH-DA fluorescence percentage (%) was estimated by recording DCFH-DA absorbance at an excitation:emission wavelength of 485:528 nm using a Synergy H1 microplate reader (BioTek, Winooski, Vermont, United States). Comparisons were made between various AE-exposed DU145 cells and the untreated control to assess the relative changes in ROS levels.

##### 2.2.1.6 Effect of ROS quencher

To reaffirm the efficacy of AE in inducing ROS-mediated oxidative stress, DU145 cells were pretreated with 10 mM N-acetyl-l-cysteine (NAC) for 2 h, followed by its exposure to AE at stated concentrations. After AE exposure for 6 h, the DU145 cells were washed and stained using DCFH-DA in the dark for 30 min. After that, the DCFH-DA fluorescence percentage (%) of each group was recorded using a spectrophotometer, as mentioned in the preceding section.

##### 2.2.1.7 Assessment of caspase/s activity

The activity levels of caspase-3 and caspase-9 were evaluated colorimetrically in AE-treated DU145 cells following the manufacturer’s instructions. Approximately 3 × 10^6^ AE-treated DU145 cells were lysed in 500 μL chilled lysis buffer followed by a brief 10 min incubation. The suspension was centrifuged at 10,000 rpm for 1 min, and the supernatant was stored on ice. Subsequently, 500 μL of the collected cell lysate from each group was allowed to react with an equal volume of reaction buffer. Eventually, DEVD-pNA (4 mM) was added to each group, and the reaction was again briefly incubated for 10 min. Each treated and untreated group’s absorbance was recorded subsequently at 405 nm.

##### 2.2.1.8 Effect of caspase inhibitors

AE-induced cytotoxicity against DU145 was also confirmed through caspase inhibitors (Ahmad et al., 2022). DU145 cells were pretreated with Z-DEVD-FMK and Z-LEHD-FMK (50 μM each; caspase-3 and caspase-9 inhibitors, respectively) for 2 h. The cells were then retreated with AE, at the abovestated concentrations for 24 h, under optimum cell culture conditions. Finally, the viability of DU145 cells was estimated through the MTT test, as stated in Section 2.2.1.2.

##### 2.2.1.9 AE-mediated effects on mitochondrial membrane potential (ΔΨm)

The AE-induced modulation of ΔΨm in DU145 was assessed colorimetrically using a mitochondrial membrane potential kit (Abcam; Cat. No. ab113852) following the manufacturer’s instruction. AE-treated DU145 cells were briefly incubated for 15 min with 50 mM carbonyl cyanide 4-(trifluoromethoxy)phenylhydrazone. The cells were then briefly retreated with tetramethylrhodamine, ethyl ester (TMRE; 1 mM) for 30 min and then washed with PBS (0.2%). Finally, the absorbance of each group was recorded at an excitation:emission of 549:575 nm.

##### 2.2.1.10 Cytochrome-c release assay

The cytosolic levels of cytochrome-c were quantified in AE-treated DU145 cells as per the protocol described previously (Tiwari et al., 2022). 1 × 10^6^ DU145 cells were exposed to the abovestated AE concentrations for 24 h. The total protein content of DU145 cells from different groups was collected using T-PER reagent (Thermo Fisher Scientific, Waltham, MA, United States) through centrifugation (1,000 rpm at 4°C for 10 min). Cytochrome-c levels within the cell lysate were quantified using an ELISA kit (Cat. No. KHO1051; Thermo Fisher Scientific, Waltham, MA, United States) by adhering to the manufacturers’ protocol.

##### 2.2.1.11 Estimation of cleaved poly(ADP-ribose) polymerase levels

AE-exposed DU145 cells were assessed for poly(ADP-ribose) polymerase (PARP) levels colorimetrically using a human-specific PARP ELISA kit (Cat. No. KHO0741, Thermo Fisher Scientific, Waltham, MA, United States) by adhering to the manufacturer’s instruction. Different AE-treated and untreated groups’ absorbance was recorded at 450 nm using a spectrophotometer (Bio-Rad, Hercules, California, United States).

##### 2.2.1.12 qRT–PCR

qRT–PCR-based studies were subsequently undertaken to assess AE-induced modulatory effects on Wnt/β-catenin signaling and other important gene targets. During the analysis, the total RNA was isolated from 1 × 10^6^ AE-exposed, and control DU145 cells, using a HiPurA TM kit (Thermo Fisher Scientific, Waltham, MA, United States). Next, cDNA was prepared through 2 μg of isolated RNA using a Verso cDNA synthesis kit (Thermo Fisher Scientific, Waltham, MA, United States) following the supplier’s manual. The sequences of primers used in the study have been included in Table 1. The β-actin gene was used as a housekeeping gene during the study, and all the normalizations were performed against this gene. Data obtained were subsequently analyzed through the comparative CT method, and the fold change in gene expression was assessed through the ^2−ΔΔCT^ method.

**TABLE 1:** List of primer sequences used for mRNA expression analysis.

Gene	Forward sequence	Reverse sequence	Ref.
β-Actin	GAA​ATC​CCA​TCA​CCA​TCT​TCC​AGG	GAG​CCC​CAG​CCT​TCT​CCA​TG	Ahmad et al. (2021)
Wnt2	TCCGAAGTAGCCGGGAAT	GAT​CGC​AGG​AAC​AGG​ACT​TTA​AT	Madueke et al. (2018)
β-Catenin	GAA​ACG​GCT​TTC​AGT​TGA​GC	CTG​GCC​ATA​TCC​ACC​AGA​GT	Wang et al. (2011)
Cyclin D1	CCGTCCATGCGGAAGATC	GAA​GAC​CTC​CTC​CTC​GCA​CT	Ahmad et al. (2021)
c-myc	AGC​GAC​TCT​GAG​GAG​GAA​CAA​G	GTG​GCA​CCT​CTT​GAG​GAC​CA	Ahmad et al. (2021)

##### 2.2.1.13 Statistical inferences

The data are the mean ± SEM of three discrete experiments performed at least thrice in triplicates. Data were analyzed through GraphPad Prism (Ver. 5.0) using one-way ANOVA and Student’s t-test as per applicability. Differences between the groups were inferred to be significant, when *p* < 0.05. * represents *p* < 0.05; **p < 0.01, and ***p < 0.001.

#### 2.2.2 *In silico* studies

##### 2.2.2.1 Docking studies

AE was docked using AutoDock Vina 4 (Wang et al., 2011). The centroid of the target protein was chosen as the binding pocket coordinate, and a grid box was placed within a cubic box of magnitude ×40 40 × 40 Å. The optimal docking position was selected from nine poses, based on the interacting residues that formed hydrogen bonds with high binding affinity (kcal/mol). The protein–ligand interactions of the docked complexes were generated using PyMol (Trott and Olson, 2010). All the figures were generated using PyMol (Lill and Danielson, 2011).

## 3 Results

### 3.1 AE impeded the growth of DU145 cells

**FIGURE 1:**
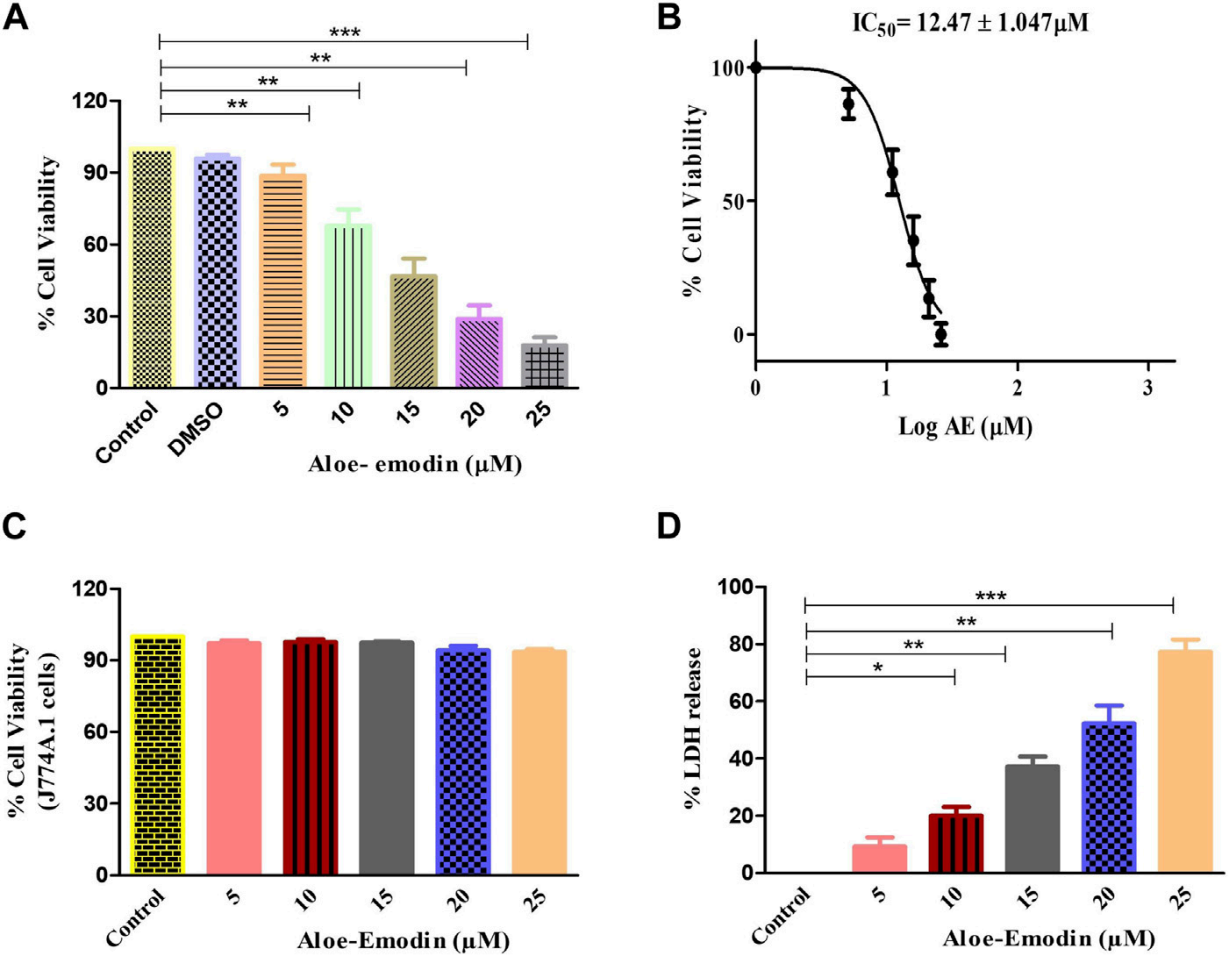
AE-instigated cytotoxic effects in DU145 cells. **(A)** Cell viability (%) of DU145 after AE exposure at a concentration range of 5–25 μM, **(B)** IC50 concentration of AE against DU145 cells, **(C)** effect of AE exposure at stated concentrations on normal murine alveolar macrophages (J774A.1) as assessed by MTT assay, and **(D)** LDH release percentage within DU145 cells upon treatment with AE. *p < 0.05, **p < 0.01, and ***p < 0.001.

To explore the plausible cytotoxic effects of AE against DU145 cells, an MTT assay was performed. It was observed that the growth and proliferation rate of the DU145 cell line was in inverse variation with the different concentrations of AE. Upon culturing of DU145 cells with AE, the growth of these cells was reduced to 88.80% ± 3.69% (5 μM), 67.75% ± 4.66% (10 μM), 46.73% ± 5.03% (15 μM), 28.92% ± 4.56% (20 μM), and 17.92% ± 2.73% (25 μM), as compared to unaltered viability in the control cells (Figure 1A). Our data, as shown in Figure 1B, exhibited that the IC50 values of AE was 12.47 ± 1.047 μM on DU145 cells, after treatment for 24 h. Therefore, based on the findings of this assay, further anti-cancer experimentation was performed. In addition, MTT assay was performed to investigate the effect of AE on normal murine macrophages J774A.1. The results showed that AE imparts insignificant cytotoxic effect on normal murine macrophages.

### 3.2 AE instigated cellular toxic effects

The cellular toxic effect of AE was further determined based on the release of LDH, an intracellular enzyme which was released in the cell culture medium when the plasma membrane gets damaged. As demonstrated in Figure 1C, the release of LDH in AE-treated DU145 cells was found to be 9.25% ± 2.59% (5 μM), 20.04% ± 2.50% (10 μM), 37.22% ± 2.81% (15 μM), 52.32% ± 4.56% (20 μM), and 77.28% ± 3.52% (25 μM) as compared to the control cells for 24 h, respectively.

### 3.3 AE-induced nuclear fragmentation

**FIGURE 2:**
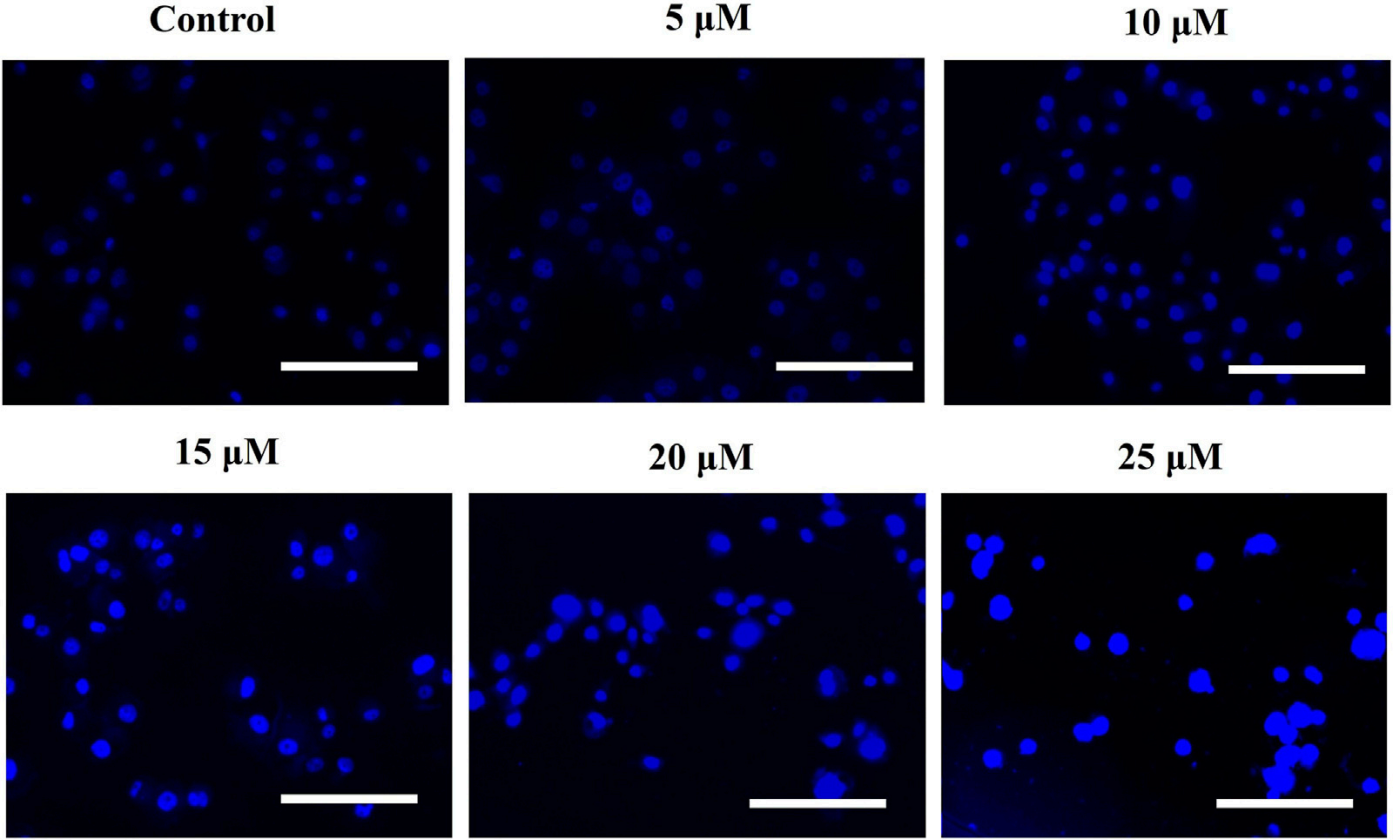
AE mediated the instigation of nuclear fragmentation, as indicated through an increased purple fluorescence in proportionality with an increase in AE concentration. Scale bar = 100 µm.

To confirm that AE-induced cytotoxic effects were due to apoptosis induction, Hoechst 33342 staining was performed. Upon treatment with varying concentrations of AE for 24 h, significant nuclear fragmentation was observed, as shown in Figure 2. The fluorescent micrographs revealed the presence of condensed and fragmented nuclei in AE-treated DU145 cells as compared to the untreated cells.

### 3.4 AE-mediated pro-oxidant activity

**FIGURE 3:**
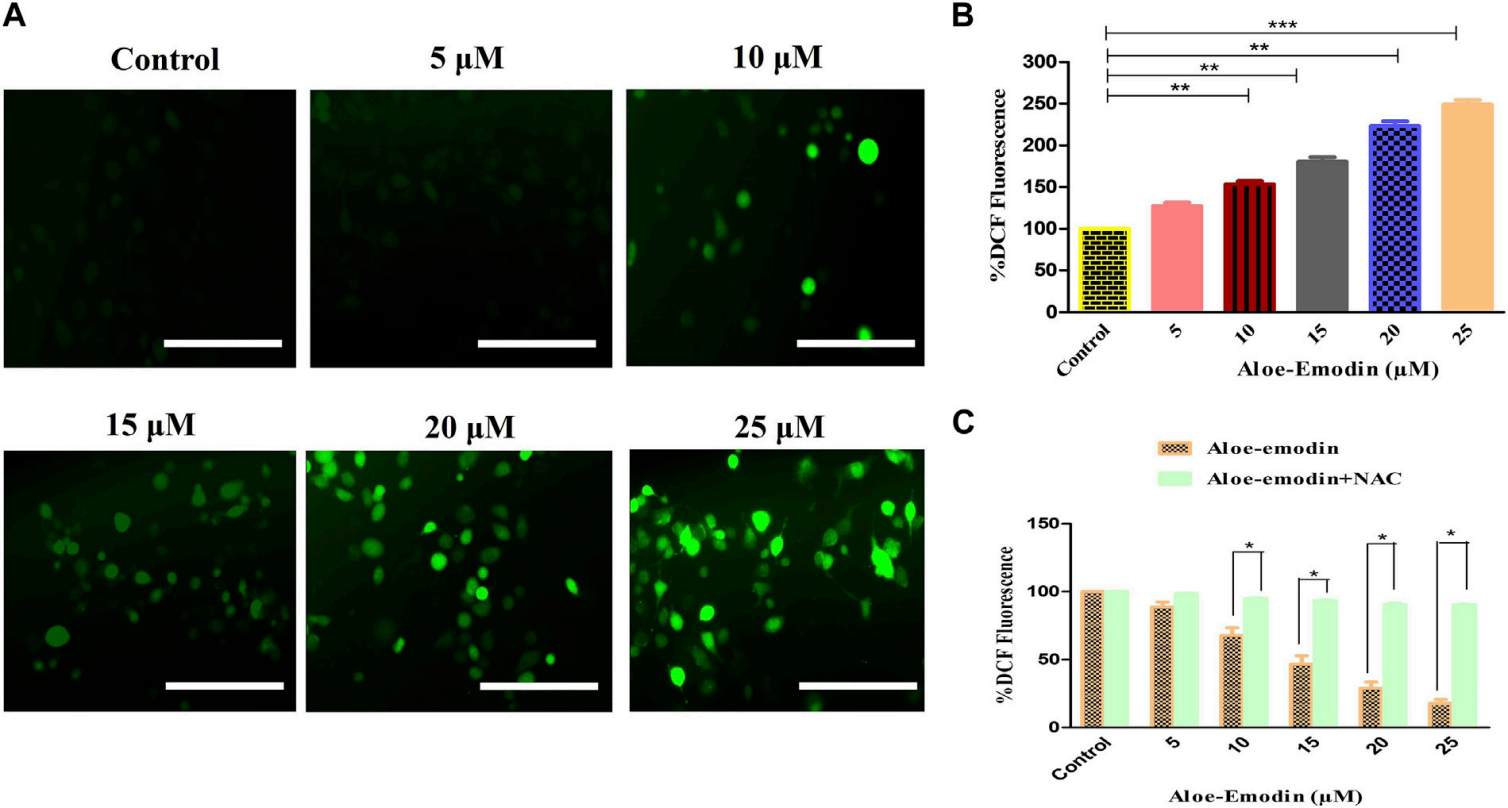
Efficacy of AE in inducing **(A)** intracellular ROS levels, **(B)** quantification of intracellular ROS levels within human androgen-independent DU145 cells, and **(C)** impaired ROS production after NAC pre-exposure in DU145 cells. Scale bar = 100 μm *p < 0.05, **p < 0.01, and ***p < 0.001.

The intracellular production of reactive oxygen species (ROS) was assessed by using DCFH-DA staining. The fluorescent micrographs of AE-treated DU145 cells, shown in Figure 3A, were captured, and the dye intensity was found to be directly proportional to the amount of ROS generated in the cells. At the indicated concentrations of 5–25 μM AE, substantial augmentation of ROS was observed in DU145 prostate cancer cells.

Furthermore, ROS’s intracellular generation was quantitatively assessed to validate our qualitative results. In the case of DU145 cells, the intracellular level of ROS was found to be 53.35% ± 2.99%, as compared to the untreated cells, at the concentration of 10 μM, which was followed by 80.42% ± 4.49% (15 μM), 123.22% ± 4.55% (20 μM), and 148.86% ± 4.44% (25 μM) as compared to the control cells (Figure 3B). These results , thus, suggested AE induced the elevation of ROS levels in a dose-dependent manner.

Moreover, to confirm that AE-treated DU145 cells mediated the generation of ROS, the amount of ROS levels in prostate cancer cells was assessed in the presence of NAC, a well-known ROS inhibitor, followed by the treatment with AE. The results demonstrated that pretreatment with NAC (5 mM) completely abolished the elevated ROS levels within DU145 cells, which verified that AE could augment the generation of ROS in prostate cancer cells (Figure 3C).

### 3.5 AE instigated caspase activation

**FIGURE 4:**
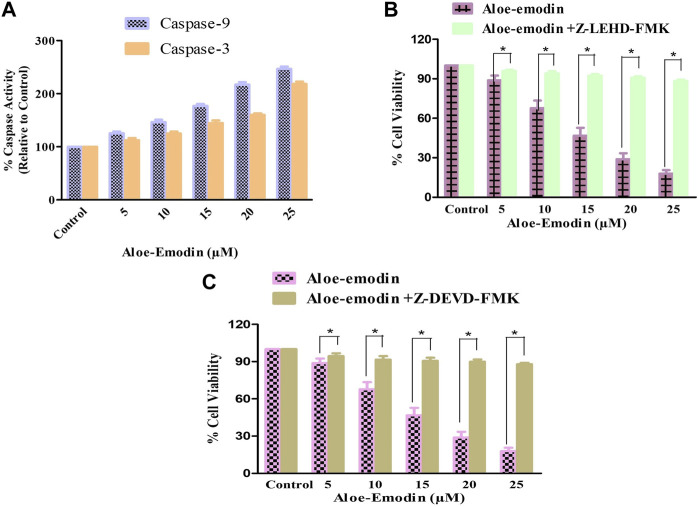
Changes in the activity percentage (%) of **(A)** caspase-9 and -3 after exposure of DU145 cells to AE and effects of specific inhibitors of **(B)** caspase-9 and **(C)** caspase-3. *p < 0.05, **p < 0.01, and ***p < 0.001.

Caspases are the proteolytic enzymes responsible for initiating the phenomenon of apoptosis. Caspase-9 initiates caspase, whereas caspase-3 is an executioner caspase, and both altogether result in apoptosis induction. As a result, it was investigated that the apoptosis induction in AE-treated DU145 cells is associated with the caspase-9 and -3 activation. Our findings demonstrated that the activities of both caspase-9 and -3 enhanced substantially, with increasing concentrations of AE, respectively. It was observed that the activities of caspase-9 increased by 25.69% ± 3.01% (5 μM), 46.70% ± 4.23% (10 μM), 77.22% ± 4.23% (15 μM), 117.29% ± 4.54% (20 μM), and 146.79% ± 4.22% (25 μM). However, the activity of caspase-3 was also increased by 12.32% ± 4.05% (5 μM), 25.23% ± 3.38% (10 μM), 44.92% ± 4.82% (15 μM), 60.00% ± 3.03% (20 μM), and 118.33% ± 4.55% (25 μM), in comparison with the control (Figure 4A). Thus, treatment with AE increased the activities of caspase-9 and -3 in a dose-dependent manner in prostate cancer cells.

**FIGURE 5:**
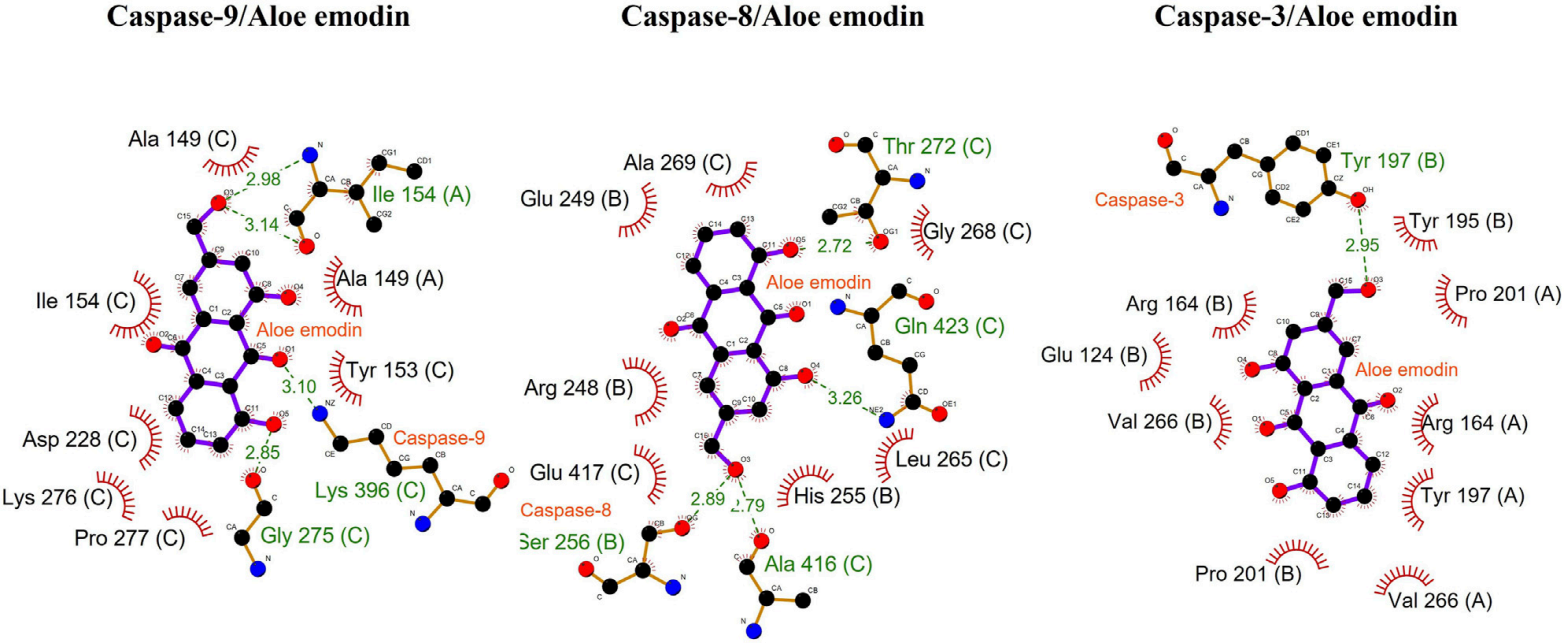
Docking analysis of caspase-9, -8, and -3 with AE.

As per the results of molecular docking analysis, it was demonstrated that the binding energy of AE toward caspase-8, -9, and -3 was −7.9, −8.3, and −7.4 kcal/mol, respectively, as shown in Figure 5A. The residues B: Arg248, B: Glu249, B: His255, B: Ala269, C: Leu265, C: Gly268, and C: Glu417 were involved in hydrophobic interactions between AE and caspase-9. However, B: Ser256 (2.89 Å), C: Thr272 (2.72 Å), C: Ala416 (2.79 Å), and C: Gln423 (3.26 Å) were found to be involved in hydrogen bonding. In case of molecular docking between AE and caspase-9, A: Ala149, C: Ala149, C: Tyr153, C: Ile154, C: Asp228, C: Lys276, and C: Pro277 residues were involved in hydrophobic interactions. Moreover, B: Ser256 (2.89 Å), C: Thr272 (2.72 Å), C: Ala416 (2.79 Å), and C: Gln423 (3.26 Å) were involved in hydrogen bonding (Figure 5B). However, the interacting amino acid residues during AE and caspase-3 interaction were A: Arg164, A: Val266, A: Tyr197, A: Pro201, B: Glu124, B: Arg164, B: Tyr195, B: Pro201, and B: Val266. However, B: Tyr197 (2.95 Å) was involved in hydrogen bonding, as shown in Figure 5C and table 2. Thus, the *in silico* results corroborated with the *in vitro* assay, and it was concluded that AE mediated the activation of caspases in prostate cancer cells.

**TABLE 2:** Binding energies of AE with caspase-9, -8, and -3.

Protein name	Binding energy 1	Binding energy 2	Binding energy 3	Binding energy average	Interacting residue hydrogen bond(s)	Interacting residue hydrophobic interactions
**Caspase-9**	−8.5	−8.6	−7.9	**−8.3**	**A:** Ile^154^ (2.98, 3.14 Å), **C:** Gly^275^ (2.85 Å), **C:** Lys^396^ (3.10 Å)	**A:** Ala^149^, **C:** Ala^149^, **C:** Tyr^153^, **C:** Ile^154^, **C:** Asp^228^, **C:** Lys^276^, **C:** Pro^277^
**Caspase-8**	−7.9	−7.9	−7.9	**−7.9**	**B:** Ser^256^ (2.89 Å), **C:** Thr^272^ (2.72 Å), **C:** Ala^416^ (2.79 Å), **C:** Gln^423^ (3.26 Å)	**B:** Arg^248^, **B:** Glu^249^, **B:** His^255^, **B:** Ala^269^, **C:** Leu^265^, **C:** Gly^268^, **C:** Glu^417^
**Caspase-3**	−7.3	−7.5	−7.5	**−7.4 ±**	**B:** Tyr^197^ (2.95 Å)	**A:** Arg^164^, **A:** Val^266^, **A:** Tyr^197^, **A:** Pro^201^, **B:** Glu^124^, **B:** Arg^164^, **B:** Tyr^195^, **B:** Pro^201^, **B:** Val^266^

Bold characters A, B, C denote respective protein chains.

### 3.6 Ameliorative effects of caspase inhibitors against AE-induced apoptosis

DU145 prostate cancer cells were treated with caspase inhibitors to ascertain that AE-mediated apoptosis induction was the result of caspase activation. The cells were pretreated with 50 µM Z-DEVD-FMK (a caspase-3 inhibitor) and Z-LEHD-FMK (caspase-9 inhibitor) for 2 h, followed by treatment with AE at the stated doses for 24 h. MTT assay was determined to measure the cell viability (Figures 4B,C). Pretreatment with caspase-3 and -9 inhibitors substantially inhibited the cytotoxic effects of AE on DU145 cells.

### 3.7 AE disrupted ΔΨm

Mitochondria are known for their contribution in inducing apoptosis. Dissipation of ΔΨm mediates apoptosis via the mitochondrial-dependent intrinsic apoptosis pathway. As demonstrated in Figures 5A,A reduction in NIR fluorescence was observed in AE-cultured DU145 after staining with Mito-NIR dye, implicating depolarization of mitochondria in comparison with the control where the ΔΨm was unaltered. Thus, AE treatment strongly altered the mitochondrial membrane potential (MMP) directly depending on AE concentration in prostate cancer DU145 cells.

### 3.8 AE mediated enhanced cytochrome-c release and PARP cleavage

To study the effect of AE on the release of cytochrome-c level in DU145 prostate cancer cells, an ELISA was performed. As demonstrated in Figure 5B, the treatment of DU145 cells with varying concentrations of AE increased the level of cytochrome-c in a dose-dependent trend. It was noted that AE treatment increased the level of cytosolic cytochrome-c by 5.66-folds at 25 μM concentration of AE. Therefore, AE-induced intrinsic or mitochondrial-dependent apoptosis via the release of cytochrome-c in the cytosol leads to apoptosis in the prostate cancer cells.

In addition, to find out whether caspase-mediated apoptosis in AE-treated prostate cancer cells was connected to PARP cleavage, cleaved PARP ELISA was performed on DU145 cells. As observed in Figure 5C, AE enhanced the level of PARP cleavage with increasing doses of AE in DU145 cells. The results demonstrated an increase in PARP cleavage levels by 2.77-folds at 25 μM concentration of AE. Therefore, AE-induced caspase-dependent apoptosis via PARP cleavage is a crucial attribute of apoptosis in prostate cancer cells.

### 3.9 AE downregulated the Wnt/β-catenin pathway

**FIGURE 6:**
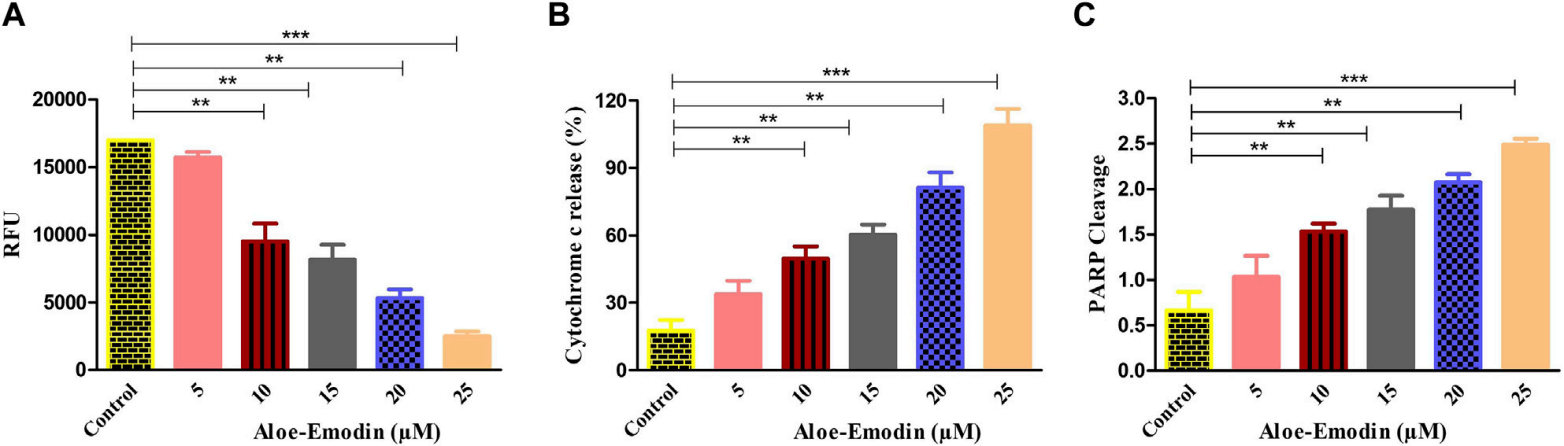
Efficacy of AE in **(A)** increasing the dissipation of ΔΨm, **(B)** elevating the release of cytochrome-c within the cytoplasm, and **(C)** enhancing PARP cleavage. *p < 0.05, **p < 0.01, and ***p < 0.001.

To further explore the mechanism by which AE regulated its anti-cancer and apoptosis effects in prostate cancer cells, we studied its effect on the Wnt/β-catenin pathway and its downstream target genes. As shown in Figure 6A, gene expression studies have substantiated that treatment with increasing concentrations of AE significantly reduced the mRNA expression of Wnt2 by 0.88 ± 0.04-, 0.67 ± 0.05-, 0.32 ± 0.03-, and 0.02 ± 0.02-folds at 5 μM, 10 μM, 15 μM, and 20 μM AE, respectively. In addition, β-catenin mRNA expression was also observed and was reduced to 0.90 ± 0.03-, 0.73 ± 0.04-, 0.52 ± 0.03-, and 0.31 ± 0.03-folds at 5–20 μM AE, respectively, compared to untreated control DU145 cells (Figure 6B).

Furthermore, the effect of AE on the downstream target genes of the Wnt/β-catenin pathway, such as cyclin D1 and c-myc, respectively, was also investigated. As shown in Figures 6C,D, it was found that AE downregulated the mRNA expression level of cyclin D1 by 0.86 ± 0.04-, 0.78 ± 0.04-, 0.43 ± 0.04-, and 0.28 ± 0.05-folds at 5 μM, 10 μM, 15 μM, and 20 μM, respectively. The expression of c-myc mRNA was elevated by 0.84 ± 0.03-, 0.70 ± 0.04-, 0.43 ± 0.04-, and 0.24 ± 0.03-folds at 5 μM, 10 μM, 15 μM, and 20 μM concentrations of AE, respectively. Thus, AE significantly downregulated β-catenin, the crucial protein of the Wnt/β-catenin signaling pathway, and expression of its downstream target proteins such as cyclin D1 and c-myc, in DU145 prostate cancer cells.

### 3.10 Molecular docking results

**FIGURE 7:**
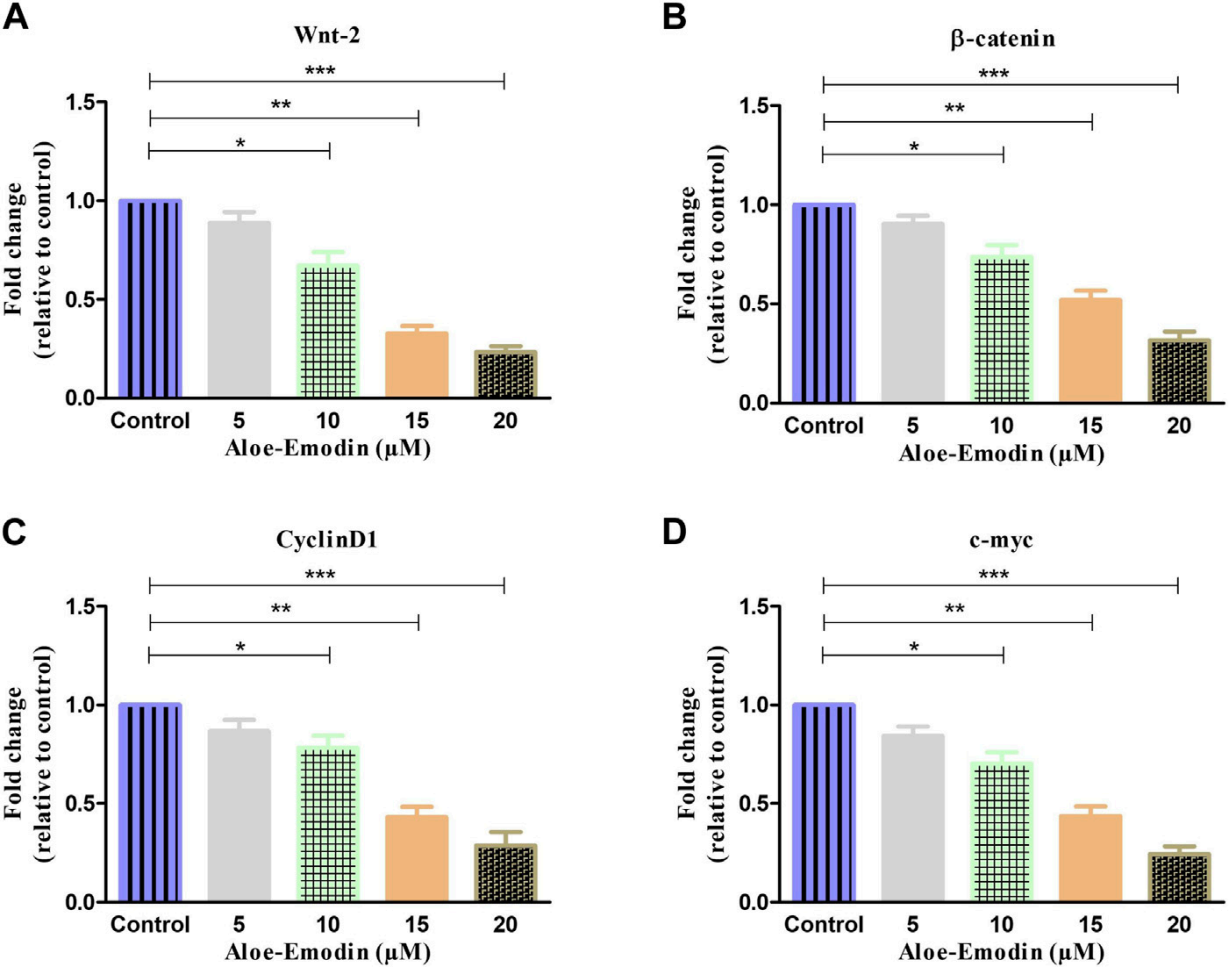
qRT–PCR-based estimation of fold change in the mRNA expression of **(A)** Wnt2, **(B)** β-catenin, **(C)** cyclin D1, and **(D)** c-myc genes. *p < 0.05, **p < 0.01, and ***p < 0.001.

**FIGURE 8:**
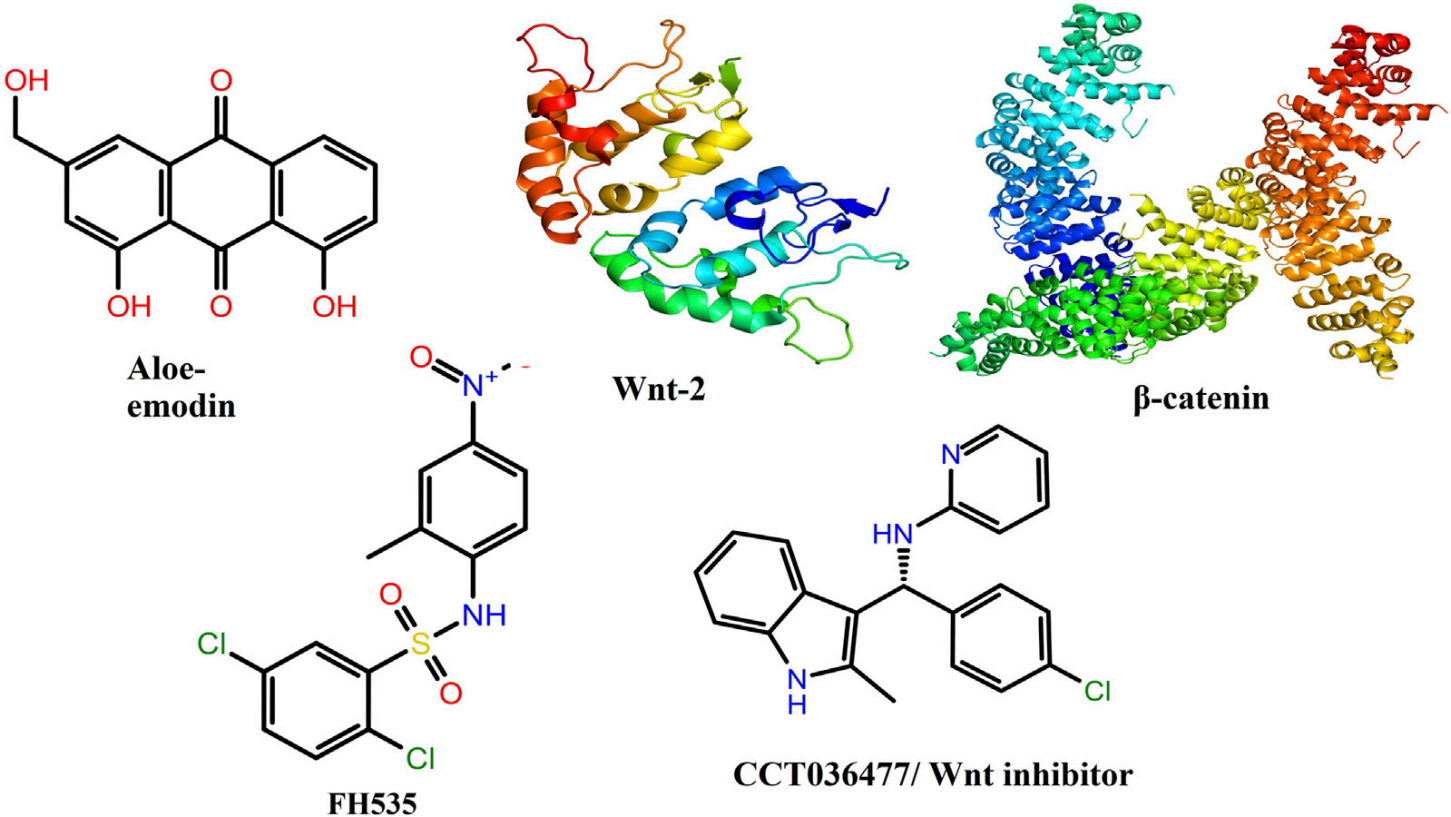
Two-dimensional and three-dimensional molecular structures of various chemicals and proteins involved in the present investigation.

**FIGURE 9:**
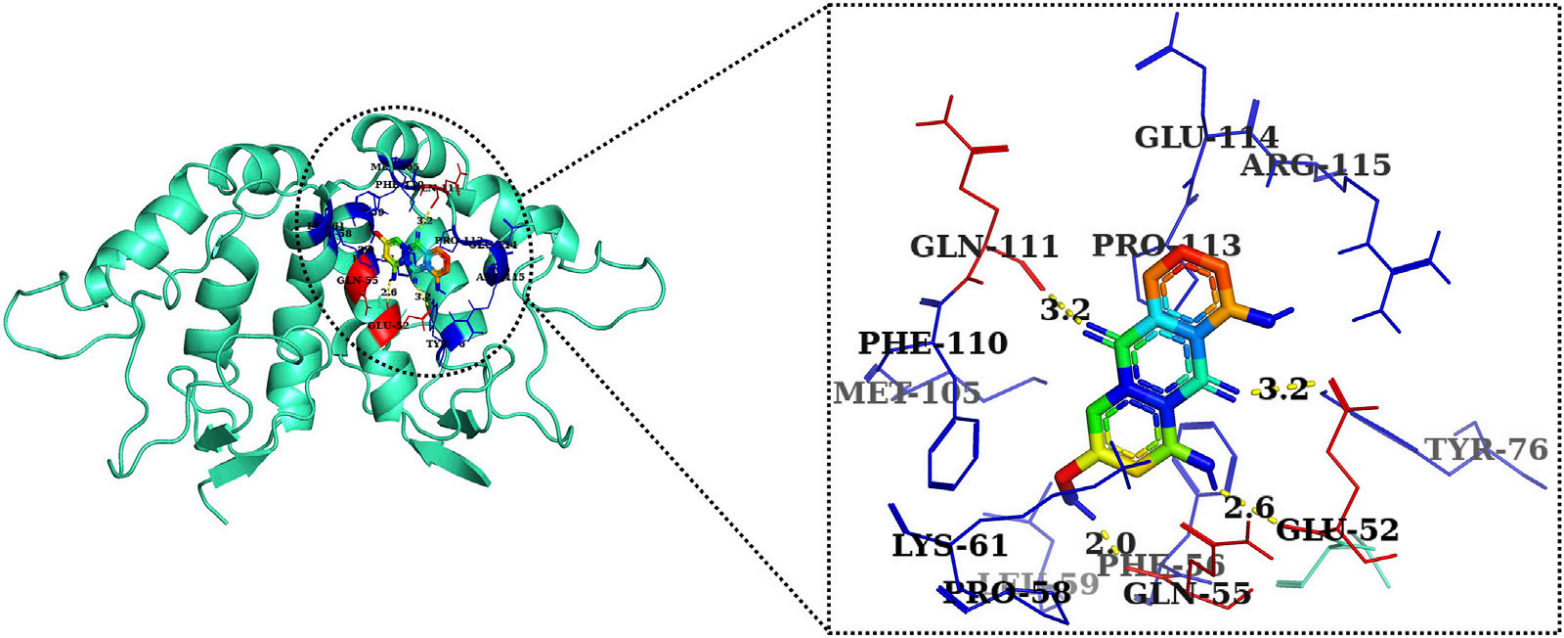
Two-dimensional interaction complex of AE with Wnt2 protein as evaluated through AutoDock Vina.

The 2D and 3D structures of AE, FH535, CCT036477, Wnt2, and β-catenin are shown in Figure 7. As per the results of molecular docking analysis, it was demonstrated that the binding energy of AE toward Wnt2 and β-catenin was −7.0 and −8.0 kcal/mol, which were comparable to the binding energies of CCT036477 and FH535 (−6.9 and −7.7 kcal/mol) as shown in Figure 8. The residues Leu59, Pro58, Lys61, Phe56, Tyr76, Met105, Phe110, Glu114, Pro113, and Arg115 were involved in hydrophobic interactions between AE and Wnt2. However, Gln111 (with a bond length of 3.2 Å), Glu52 (with bond lengths of 2.6 and 3.2 Å), and Gln55 (with a bond length of 2.0 Å), and other residues were found to be involved in hydrogen bonding. However, in case of molecular docking between Wnt2 and CCT036477 (Wnt2 inhibitor), Asp29, Ile30, Val60, Lys61, Gln63, Cys64, Glu67, Leu68, Arg69, and Phe70 residues were involved in hydrophobic interactions. Moreover, Pro66 (bond lengths 2.3 Å) and Ser65 (bond length 2.2 Å) were involved in hydrogen bonding (Figure 9).

**FIGURE 10:**
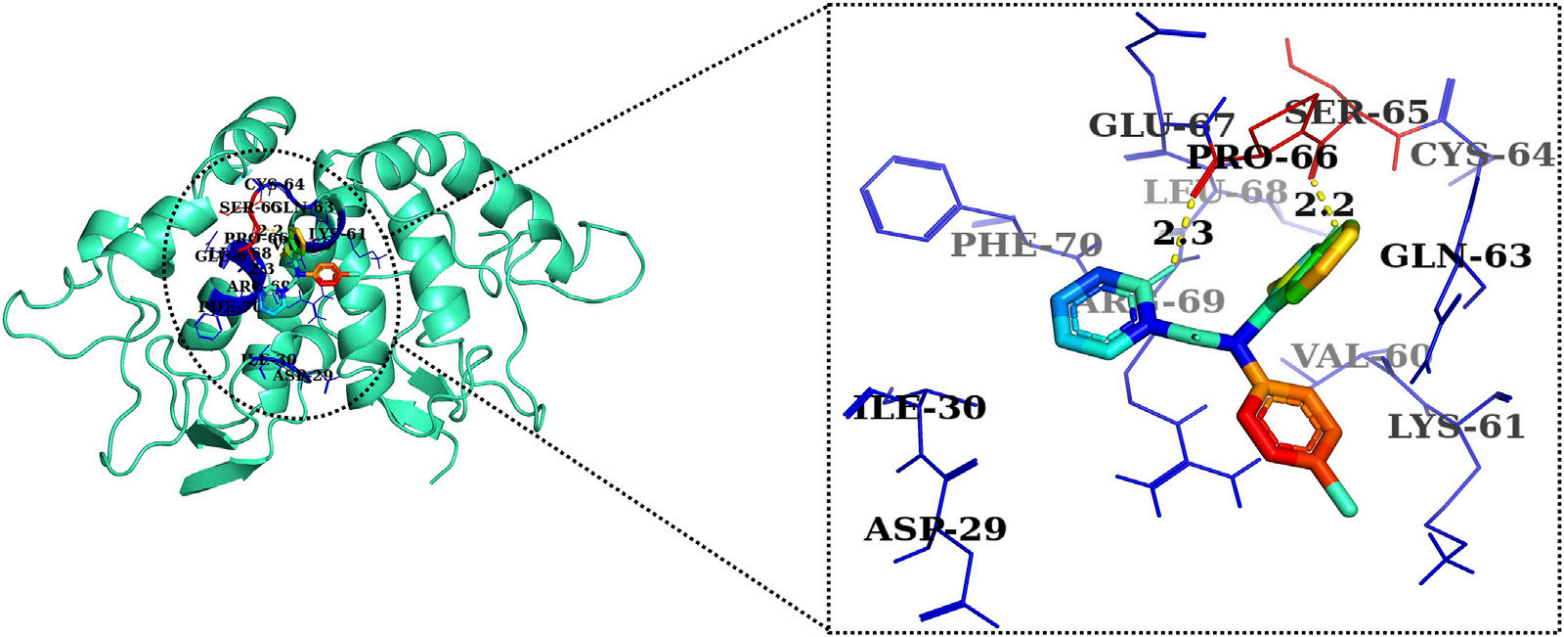
Two-dimensional interaction complex of the CCT036477 inhibitor with the Wnt2 protein complex as evaluated through AutoDock Vina.

**FIGURE 11:**
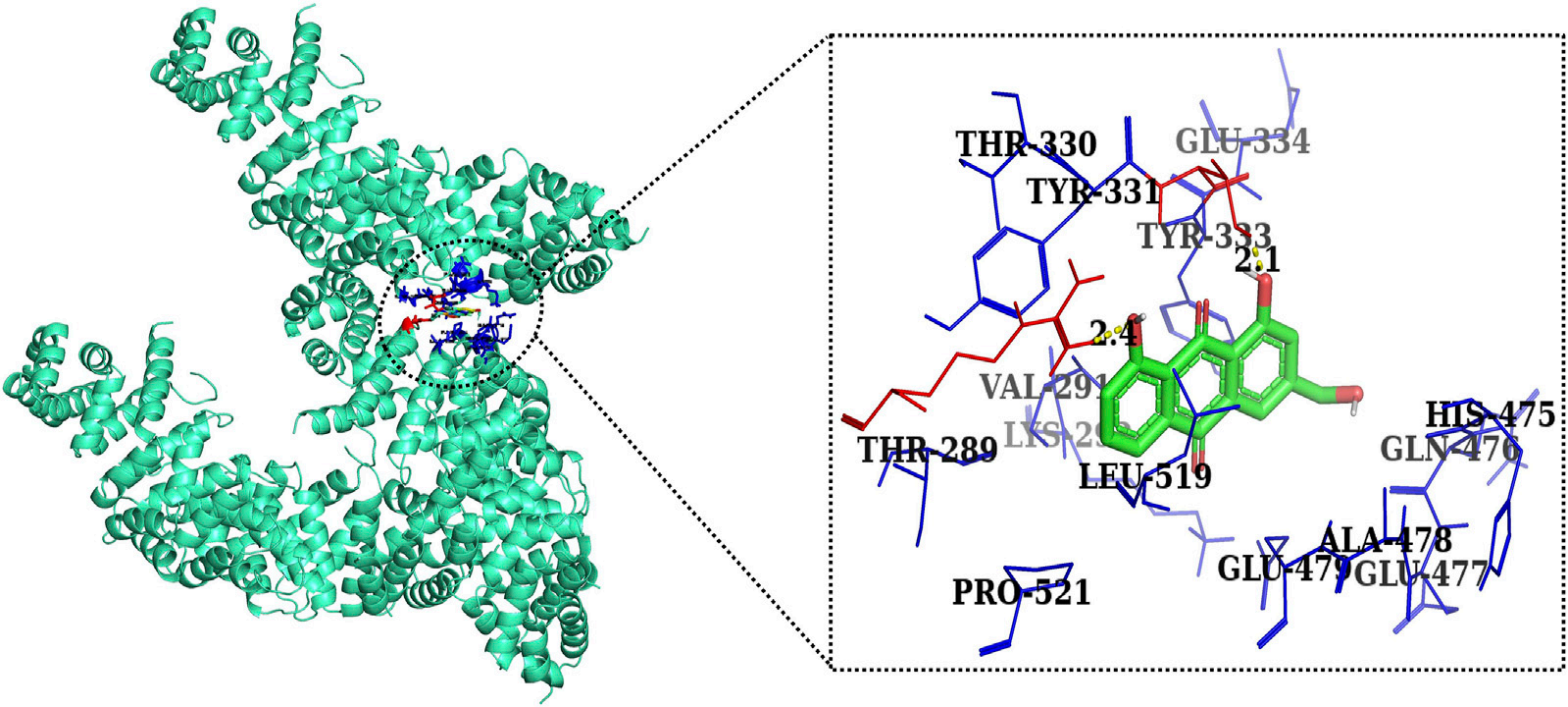
Two-dimensional interaction complex of AE with the β-catenin protein complex as evaluated through AutoDock Vina.

Furthermore, in case of molecular docking between β-catenin and AE, Thr330, Tyr331, Glu334, Tyr333, Leu519, Val251, Lys292, Thr289, Pro521, Glu479, Ala478, Glu477, Gln476, and His475 residues were involved in hydrophobic interactions. However, Thr332 (bond length 2.1 Å) and Arg582 (bond length 2.4 Å) residues were involved in hydrogen bonding. However, in the case of molecular docking between β-catenin and FH535 (β-catenin inhibitor), residues as shown in Figure 10, namely, Arg582, Leu519, Gln479, Gln482, Ala478, Ser473, Thr472, Arg474, His475, Glu334, Tyr333, Tyr331, Lys292, and Val291 were involved in hydrophobic interactions. However, there was one residue, Thr332 (bond length 2.3 Å), which was involved in hydrogen bonding (Figure 11). Thus, AE is explored as a propitious natural compound with efficacy to Wnt/β signaling through molecular docking studies. The binding energies of AE with breast cancer targets (Wnt2 and β-catenin) and the interacting amino acids are summarized in Table 3.

**TABLE 3:** Binding energies of AE with Wnt2 and β-catenin in comparison with their respective standards CCT036477 and FH535.

Compound	Binding energy (Kcal/mol)	Hydrogen bonds	Hydrophobic interactions
Wnt2 + AE	−7.0	Gln111, Glu52, and Gln55 and other residues were found to be involved in hydrogen bonding	Leu^59^, Pro^58^, Lys^61^, Phe^56^, Tyr^76^, Met^105^, Phe^110^, Glu^114^, Pro^113^, and Arg^115^
Wnt2 + CCT036477	−6.9	Pro66 and Ser65 are involved in hydrogen bonding	Asp29, Ile30, Val60, Lys61, Gln63, Cys64, Glu67, Leu68, Arg69, and Phe70
β-Catenin + AE	−8.0	Thr332 and Arg582 residues are involved in hydrogen bonding	Thr330, Tyr331, Glu334, Tyr333, Leu519, Val251, Lys292, Thr289, Pro521, Glu479, Ala478, Glu477, Gln476, and His475

## 4 Discussion

Despite the significant modern-day progress, the usage of present therapeutic modalities, including the standard radio and chemotherapeutics, needs to be improved by the systemic cytotoxicity and generation of resistance toward these therapies (Liu et al., 2021). This has necessitated the usage and exploration of efficacious natural compounds for their plausible anti-cancer role applicable to human use. Indeed, previous reports have shown that AE possesses a wide array of pharmacological attributes, including anti-bacterial and anti-microbial activities (Dong et al., 2020a). However, of all these attributes, the intrinsic anti-cancer efficacy of AE is of particular interest. Previously, the anti-cancer effects of AE have been reported in colon, breast, pancreatic, and lung cancer cells (Sanders et al., 2018). Indeed, it has been demonstrated that AE modulates key signaling pathways, including PI3K/Akt/mTOR, ROS-JNK, MAPKs, Ras/ERK, and PKC (Acevedo-Duncan et al., 2004; Tu et al., 2016; Tseng et al., 2017; Dou et al., 2019; Shen et al., 2020). Furthermore, AE is also associated with regulating the expression levels of key genes, namely, c-Myc, NF-κB, casein kinase II, and ALP (Chen et al., 2014; Dong et al., 2020a). Nevertheless, the mechanistic insight into the functioning of AE in androgen-independent DU145 prostate cancer cells is still vague. The results from our study indicated that AE indeed exerted significant cytotoxic effects on DU145 cells. Based on the observations of MTT and LDH release assay, the present study showed that AE impeded the proliferation of DU145 cells in direct proportion to the concentration of AE.

To gain a better insight into the mechanistic anti-cancer effects of AE, its functioning at molecular levels impeding the viability of DU145 cells was subsequently investigated. Oxidative stress mediated by ROS plays a major role in varying the redox state of cancer cells and is, thus, an important target for various anti-cancer therapeutics (Liou. and Storz, 2010). ROS levels subsequently serve as a critical impetus for activating various signaling pathways in regulating key intracellular processes such as inflammation, cell proliferation, migration, and apoptotic cell death (Ye et al., 2016). Due to its important role in cancer cells, the investigators studied AE-mediated effects on ROS levels in DU145 cells. The results indicated that upon AE exposure, intracellular ROS levels escalated considerably in the prostate cancer cells, which was characteristically proportional to AE concentration. This escalation of ROS levels could further be correlated with the activation of mitochondria-dependent apoptotic cell death. Intriguingly, AE exposure in ROS quencher pretreated DU145 cells significantly reduced ROS generation, which was coherent with our observation that AE induced the escalation of ROS generation in DU145 cells.

Being an important member of the cysteine protease family, caspases play prerequisite roles in modulating the apoptotic pathway (McIlwain et al., 2013). Caspase activation during the onset of apoptosis is primarily mediated by the death-receptor pathway, where caspase-8 is established to provide key impetus. Furthermore, caspase-8 activation results in the downstream impelling of the mitochondria-dependent apoptotic pathway due to the concomitant cytosolic accumulation of cytochrome-c (Tummers and Green, 2017). Importantly, these pathways in their activated state led to the activation of caspase-3 by cleavage-dependent pathways. The observations from the present study indicated increased ROS levels concomitantly following the onset of apoptotic pathways after AE exposure, dissipated ΔΨm followed by increased caspase activities, and modulated expression of apoptosis-associated proteins. Thus, it can be inferred that AE-induced apoptotic cell death in DU145 cells was significantly modulated by caspase activation. Intriguingly, AE-mediated cytotoxicity was considerably alleviated in the presence of caspase-3 and -9 inhibitors, indicating the relevance of these caspases in impelling apoptotic cell death.

In mammalian cells, mitochondria are important sites mediating ROS generation, resulting in dysfunctional mitochondria and the dissipation of ΔΨm, leading to the discharge of cytochrome-c from mitochondria (Zorova et al., 2018). Indeed, in the present investigation, a significant decline in ΔΨm was observed in DU145 cells after exposure to AE. This observation supported the notion that dissipated ΔΨm activates caspase-3 by functionally activating endonuclease. This dissipation of ΔΨm after AE exposure corroborated with the ROS levels and mitochondria-dependent apoptosis. Intriguingly, initiating apoptotic cell death in cancers is considered an attractive target for therapeutic intervention (Arfin et al., 2021). Among various other characteristics, DNA fragmentation and chromatin condensation represent the major characteristic during the onset of apoptosis. Our observations were indeed in line with the stated notion. During the study, it was inferred that AE exposure resulted in the apoptotic cell death of DU145 cells on the basis of observations recorded during the DAPI assay. Furthermore, this notion was also corroborated by our findings during PARP cleavage and cytochrome-c release assay in DU145 cells.

**FIGURE 12:**
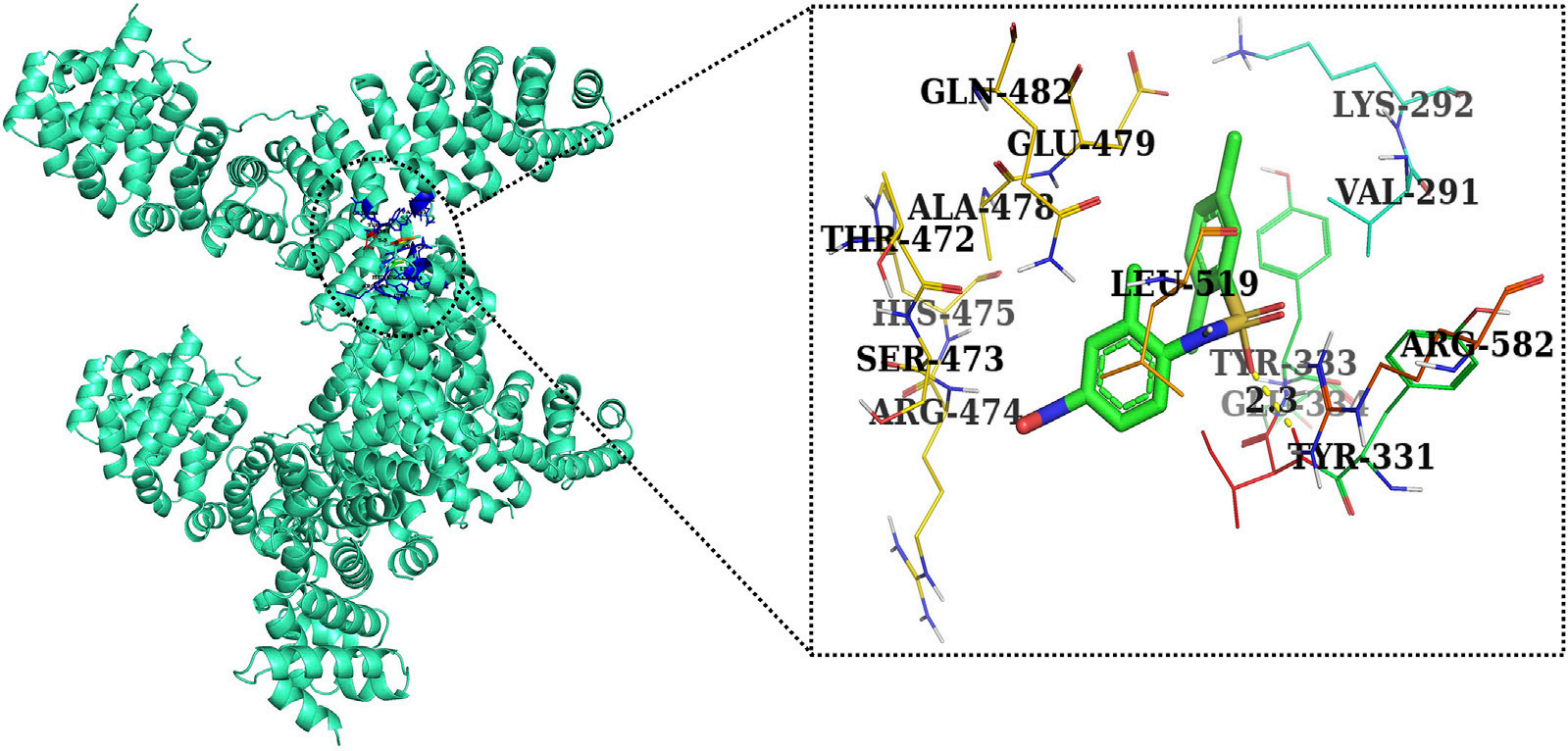
Two-dimensional interaction complex of the FH535 inhibitor with the Wnt2 protein complex as evaluated through AutoDock Vina.

Developmental signaling pathways such as the Wnt pathway are indispensable in regulating proliferation, the initiation of drug resistance, and pathogenesis in prostate carcinoma (Seo et al., 2017). Reports have substantiated that the abnormal expression of Wnt receptors, their ligands, and inhibitors plays a significant role in the onset of prostate cancer (Zhu et al., 2020). The present study also observed that AE-induced impaired DU145 cell growth correlated with altered Wnt/β-catenin signaling. The study showed that AE reduced the Wnt2 and β-catenin expression. Intriguingly, AE also succeeded in lowering cyclin D1 mRNA, which subsequently promotes the progression of cells through the G0/G1 checkpoint (Behrens, 2000; Fu et al., 2004). Similarly, survivin is another important downstream target of Wnt/β-catenin signaling and is also an important member of anti-apoptotic proteins.

c-Myc is a multifaceted oncogene that regulates various tumorigenesis-, proliferation-, and cell growth-related processes in several carcinomas. Among these, promoting the progression of cells to different phases of cell cycle is the most important biological function of c-Myc. Indeed, AE also exhibited its competence in deflating the mRNA expression of c-Myc in DU145 cells. This finding subsequently indicated that the impairment of c-Myc levels could plausibly be related to the onset of apoptotic cell death and arrest of cell cycle progression in prostate carcinoma. In addition, *in vitro* findings were subsequently corroborated by performing *in silico* studies. AE was docked with Wnt2 and β-catenin at the binding energies of −7.0 and −8.0 kcal/mol, which was comparable to the binding energies of CCT036477 and FH535 (−6.9 and −7.7 kcal/mol) (Figure 12). Therefore, correlation was performed between *in silico* findings and qPCR results so as to establish that AE downregulated the Wnt/β-catenin signaling pathway and exhibited strong binding affinity toward Wnt2 and β-catenin. Furthermore, these results are in support of real-time PCR results and provide a strong rationale that AE significantly inhibited the Wnt/β-catenin signaling in prostate cancer cells. In total, in the current investigation, it was reported that AE modulated the Wnt/β-catenin pathway and inhibited the growth of androgen-independent prostate cancer cells. Thus, the findings from this research could provide a novel insight into the involvement and the underlying molecular mechanism of AE in prostate cancer.

## 5 Conclusion and future perspective

The present investigation indicated the anti-proliferative efficacy of AE by modulating the important components and targets of Wnt/β-catenin signaling in androgen-independent DU145 prostate cancer cells. AE was also competent in instigating apoptotic cell death, subsequently exerting anti-cancer effects on DU145 cells. The observations from the present report indicate the plausible usage of AE as an anti-cancer intervention in prostate cancer treatment. AE can also be further investigated as plausible adjunct therapeutics with the existing chemotherapeutic drugs in treating prostate cancer. Moreover, AE was mostly tested on a limited range of cancer cell lines, so its spectrum of activity needs to be expanded. In certain instances, the effectiveness of AE was limited by its poor bioavailability. Therefore, researchers should lay emphasis not only on the efficacy of compound, which is of substantial interest, but also on effective drug delivery systems aimed at overcoming the pharmacokinetic issues along with studying derivatives with a high degree of biological efficacy and availability.

## Data Availability

The original contributions presented in the study are included in the article/Supplementary Material; further inquiries can be directed to the corresponding author/s.
